# A novel efficient bioflocculant QZ-7 for the removal of heavy metals from industrial wastewater

**DOI:** 10.1039/c9ra04683f

**Published:** 2019-09-04

**Authors:** Zayed M. M. Abu Tawila, Salmah Ismail, Salem S. Abu Amr, Emad K. Abou Elkhair

**Affiliations:** Institute of Biological Science, Faculty of Science, University of Malaya 50603 Kuala Lumpur Malaysia salmah_r@um.edu.my +60379672143; Biology Department, Faculty of Science, Al-Azhar University Gaza Palestine; Malaysian Institute of Chemical & Bioengineering Technology, Universiti Kuala Lumpur, (UniKL, MICET) 78000 Melaka Malaysia

## Abstract

In this study, a novel bioflocculant QZ-7 was produced from *Bacillus salmalaya* 139SI for industrial wastewater treatment. Biochemical analysis, FTIR, scanning electron microscopy-energy dispersive X-ray spectroscopy, and thermogravimetric analysis were performed. A synthetic wastewater sample was used to validate the performance of the prepared OZ-7 for the adsorption efficiency of As, Zn^2+^ Pb^2+^, Cu^2+^, and Cd^2+^ under optimal experimental conditions such as initial metal concentrations, pH, contact time (h) and QZ-7 adsorbent dosage (mg mL^−1^). The maximum removal efficiency for Zn^2+^ (81.3%), As (78.6%), Pb^2+^ (77.9%), Cu^2+^ (76.1%), and Cd^2+^ (68.7%) was achieved using an optimal bioflocculant dosage of 60 mg L^−1^ at 2 h shaking time, 100 rpm and pH 7. Furthermore, the obtained optimum experimental conditions were validated using real industrial wastewater and the removal efficiencies of 89.8%, 77.4% and 58.4% were obtained for As, Zn^2+^ and Cu^2+^, respectively. The results revealed that the prepared bioflocculant QZ-7 has the capability to be used for the removal of heavy metals from industrial wastewater.

## Abstract 2

In this study, a novel bioflocculant was produced using *Bacillus salmalay*a 139SI for industrial waste water treatment.

## Graphical abstract

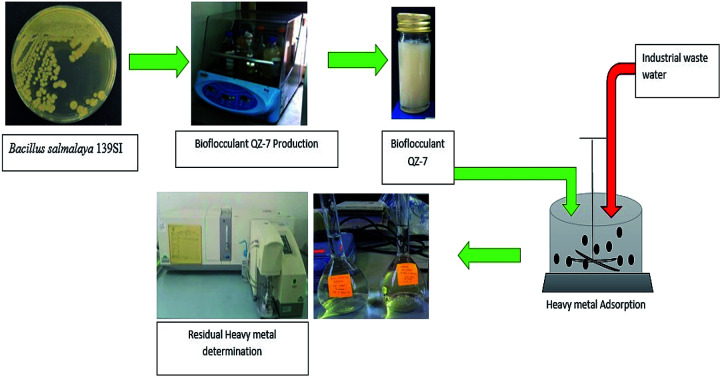

## 1. Introduction

Resources of fresh drinking water are globally considered as the most significant reservoirs. For survival, fresh drinking water must be accessible to all living organisms on the planet.^1,2^ Increasing world population, developing industries, and long droughts decrease the available water resources.^3^ Inefficient treatment of industrial wastewater results in the contamination of natural water resources by high level of refractory pollutants.^4^ Metals are the most abundant chemical elements found in the earth's crust; they are also frequently found in foods at low concentrations. Thus, their nutritional or toxicological hazard differs according to the types of metals and their concentrations.^5^ Anthropogenic industries, such as agriculture and mining, lack environment-friendly techniques and along with natural disasters, such as earthquakes, storms, and volcanoes, discharge toxic substances in water.^6^ Wastewater pollution containing heavy metal ions has raised a wide concern for environmental sustainability, calling for the development of high-efficiency and low-cost materials for wastewater treatment.^7^ These contaminants are characterized into three main groups: organic, inorganic, and biological elements.^6^ Waste production from mines results in vast environmental complications, in which draining from tailings and rock waste discharges can increase the concentration of acid and noxious heavy metals. Heavy metals persist in trace quantities in the environment and at small concentrations, they play significant roles in the living organisms; however, they can cause toxicity if their concentrations exceed the recommended level. Toxic metal contamination is considered as one of the pivotal environmental problems, which is generally prevalent in industrialized cities, where the increase in the toxic metal concentration beyond the recommended level can increase many health problems such as carcinogenic diseases and Alzheimer's or Huntington's disease.^7^ For example, heavy metals such as arsenic, lead, mercury, copper, cadmium, nickel, zinc, and chromium act as the primary noxious wastes in fresh water reservoirs because of their harmful, non-biodegradable, and persistent nature.^3^ Industrial development is the basic source of heavy metals that pass through various environment paths, including water, soil, air, and the biosphere. Heavy metals are easily absorbed by seafood, vegetables and eventually by the human body because of their high solubility in the aquatic environment.^3^ The combination of heavy metals with the sulfhydryl groups of proteins prevents enzyme activity, and such combination occurs in contaminated ecosystems, leading to consequences for the human health.^8^ Thus, in living organisms, the strong affinity of arsenite to methanethiol (mercaptan) groups, such as peptides, proteins, and amino acids, involves the types of enzymes that can cause severe noxiousness to humans.^9^ Environmental systems (abiotic methods) have been utilized for removing heavy metals from polluted locations. Abiotic methods include adsorption, the use of activated carbon, reverse osmosis, chemical precipitation, and ion exchange. Flocculating materials are commonly applied in numerous fields, including water treatment, fabric industry, cosmetics, drug treatment, and those related to fermentation development.^10^ In literature, several applications and techniques have been reported for industrial wastewater treatment. Several specialists have worked on advanced competent treatment skills. Treatment technologies are principally based on physicochemical, advanced oxidation, or electrochemical methods.^4^ Recently, a biological remediation technique using a permeable reactive barrier (PRB) has been identified as the most cost effective and safe technique; it involves organic, (date seed, *Moringa oleifera* and wood chips) and inorganic (limestone; CaCO_3_) carbonaceous materials in varying proportions for nitrate (NO_3_) remediation.^11^ Phytoremediation is a potential cost-effective technology for remediating heavy metal-contaminated soils. For example, *Pennisetum* sp. was found to be the best species for Cu and Cd removal.^12^ Also, the biomasses of *Elsholtzia splendens* and *Sedum plumbizincicola* were found to be good species for Cu and Cd removal.^12^ The obtained results showed that the functionalized halloysite nanotubes enhanced the adsorption capability of the clay mineral and it made nanoclay a good candidate for heavy metal removal from aqueous solutions.^13^ Physicochemical procedures comprise adsorption, ion exchange, membrane filtration, and chemical precipitation. Electrochemical methods are characterized by electro-coagulation, electro-deposition, and electro-flotation.^3^ Photo-catalysis is a unique feature of innovative oxidation techniques. Recently, nanotechnology has been applied in the treatment of wastewater. Among these potential approaches, those with cost-effectiveness, environment-friendliness, and no extra contaminant features are favored.^3^ Flocculant agents are categorized into three groups: organic polymer flocculants, such as polyacrylamide derivatives and polyacrylic acid; inorganic flocculants, such as polyaluminum chloride and aluminum sulfate; and microbial bioflocculants, which include glycoprotein, polysaccharides, and proteins.^14,15^ Wang *et al.* (2011)^16^ reported that the flocculants adsorbed onto the surface of suspended particles are essential for the application of vigorous attractive forces to control electrostatic repulsive force. Flocculating methods have an immediate effect on the suspended particle size, that is, a larger size results in more rapid settling down of particles.^17^

Zhang *et al.* (2014)^18^ reported that the selection of the flocculant type presents a significant effect on the capacity of the flocculating techniques, potency of the accumulated particles, and the degree of bond stability as a consequence of the flocculation process. However, regardless of the significant effectiveness of the flocculating practices in water treatment, the main disadvantage of flocculation is the production of breakable flocs at low temperatures; these flocs can scatter the applied physical force.^1^

Therefore, “green technology” bioflocculants have attracted increasing methodical and technical consideration in environmental wastewater treatment because they are non-hazardous to humans, possess biodegradable constituents, and become free from pollutants by intermediate deprivation.^19^ In addition, bioflocculants have been generally involved in different applications, such as cleaning up of wastewater, decontamination of organic substance components from sugar, dewatering, dredging, pulp and paper industry, and condensation of mine wastes.^14,20^ A high cost prevents the advancement of bioflocculants for marketable use.^18,21^ Therefore, industries of large-scale production and utilization of bioflocculants as prospective alternatives of their artificial counterpart remain to be achieved. The present work aimed to evaluate the potential capability of a polymeric bioflocculant QZ-7 in removing heavy metals from industrial wastewater. In this research, the bioflocculant QZ-7 was produced from *Bacillus salmalaya* strain 139SI.^22^

Different bioflocculant dosages, initial heavy metal concentrations, pH values, and reaction times were examined and evaluated using synthetic wastewater samples. The performances obtained under the optimal operational conditions were compared with those of the actual wastewater samples.

## 2. Material and methods

### 2.1 Materials

Bioflocculant QZ-7 was produced from *B. salmalaya* 139SI.^22^ Heavy metal compounds including arsenite, lead acetate, copper sulfate, cadmium chloride, and zinc sulfate were used to prepare heavy metal stock solutions, and they were of analytical grade. Solutions of 1 M of HCl and NaOH were used for pH adjustment. Nitric acid was used for digestion process. Industrial wastewater samples were obtained from the Perlis state of Malaysia. Inductively coupled plasma mass spectrometry (AAS; Model AA 6300, Shimadzu, Japan) was performed to determine heavy metal concentration before and after adsorption. pH of the prepared solution was measured through a digital pH meter (BP3001 Trans instrument) using a calibrated electrode with standard buffer solutions.

### 2.2 Characterization of bioflocculant QZ-7

#### 2.2.1 Biochemical analysis

A total of 2.74 g of purified QZ-7 was obtained from 1 L of fermented broth. The pure bioflocculant solution was analyzed using the anthrone method for total carbohydrate content, expending glucose as the standard solution, whereas the carbazole-sulfuric acid method was used to determine uronic acid according to the work of Chaplin and Kennedy.^23^ Bradford assay was performed with bovine serum albumin as the standard solution to determine the total protein quantity.^24^

#### 2.2.2 Fourier-transform infrared spectroscopy (FTIR)

QZ-7 sample was characterized using FTIR spectroscopy (PerkinElmer spectrum 400) to determine the type of functional group. The dried pure bioflocculant sample was mixed with potassium bromide (KBr) powder and hard-pressed into pellet discs. FTIR spectra were triplicated and determined in the frequency range of 4000–400 cm^−1^ under ambient conditions,^25^ while the image resolution was set at 600 dpi.

#### 2.2.3 Scanning electron microscopy-energy dispersive X-ray (SEM-EDAX)

SEM images were obtained at 2 kV (HITACHI-SU8220, Japan). EDX measurements were obtained through an X-ray detector and examined using the Aztec 22650005761133539 software.

#### 2.2.4 Thermogravimetric analysis (TGA)

Approximately 10 mg of purified QZ-7 was examined using a TGA analyzer (PerkinElmer TGA 4000) at the temperature range between 50 °C and 800 °C and at a heating degree of 10 °C min^−1^ in a nitrogen gas flow rate of 20 mL min^−1^.

### 2.3 Factors affecting bioflocculant performance

#### 2.3.1 Effect of bioflocculant dosage on flocculation efficiency

The optimal concentration of QZ-7 was investigated. A jar-test was used for the clarifying kaolin clay suspension (4 g L^−1^) at a neutral pH of 7. Various dosages of pure bioflocculant (10 mg L^−1^) were used (0.1 mg mL^−1^ to 6 mg mL^−1^). The samples of kaolin clay suspension mixtures containing 100 mL (4 g L^−1^, pH 7) of bioflocculant from 0.1 mg mL^−1^ to 6 mg mL^−1^ concentrations and 3 mL of 1% CaCl_2_ were stirred vigorously and kept standing for 2–5 min. Calcium ions were added to neutralize the negative repulsion charges for facilitating enhanced flocculation. Optical density (OD) of the clarified solution was tested at 550 nm. A control experiment was also organized, and the bioflocculant QZ-7 solution was replaced with distilled water. Flocculating activity was estimated according to the following eqn (1):^26^1
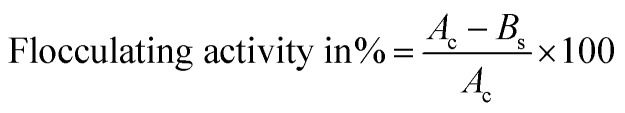
where, *A*_c_ and *B*_s_ are the control sample and OD at 550 nm, respectively.

#### 2.3.2 Effect of pH on flocculating efficiency

To evaluate the effect of pH on the flocculating activities of QZ-7, the pH variation of the kaolin suspensions was adjusted between 2 and 11 using HCl or NaOH. The significance of using a wide range of pH measurements was to regulate the condition that bears the flocculation process taking place by the support of the bioflocculant and obtain an optimal range where it best performed. A control experiment was also organized, and the bioflocculant QZ-7 solution was replaced with distilled water. Flocculating activity was estimated according to the above eqn (1).^26^

#### 2.3.3 Cation effects on flocculating activities of bioflocculant QZ-7

The influences of different cations on the flocculation activity of QZ-7 were studied by the addition of CaCl_2_, KCl, NaCl, LiCl, MnCl_2_, MgCl_2_, AlCl_3_ and FeCl_3_ at a concentration of 1 mM. Flocculating efficiency was also measured.^27^ The measurement of flocculating activities was conducted in the same manner as described in Section 2.3.1.

### 2.4 Factors affecting heavy metal adsorption

Removal of heavy metals using a bioflocculant was measured according to the work of Lin and Harichund.^28^ The metal salts used comprised copper sulfate, lead acetate, sodium arsenate, zinc sulfate, and cadmium chloride (Sigma Co). The effects of different initial concentrations of heavy metals (20, 40, 60, 80, and 100 mg L^−1^), bioflocculant concentrations (20, 40, 60, 80, and 100 mg L^−1^), and pH values (3, 5, 7, and 9) on metal adsorption were examined. QZ-7 solutions (5 mL) were added through a dialysis tube in flasks having 100 mL of each metal-salt solution and kept for 24 h at room temperature with shaking at 100 rpm. Next, 2 mL of each solution was filtered through an Amicon filter (Centrifree) and then acidified with 1% nitric acid solution for residual metal determination. The metal amounts removed from the samples tested, that is, the bound polymers were measured previously, whereas those that remained after 24 h were detected using inductively coupled plasma mass spectrometry (AAS; Model AA 6300, Shimadzu, Japan); the removal percentage rate for each element was calculated.^2^ Control (5 mL of deionized water) solutions were also prepared in the dialysis tube for different metal-salt solutions. Removal percentage (*R*) is expressed by eqn (2):

2 *R* = ((*C*_i_ − *C*_e_)/*C*_i_) × 100

Here, *C*_i_ and *C*_e_ correspond to the original and equilibrium metal concentrations.

### 2.5 Removal of heavy metals from industrial wastewater using bioflocculant QZ-7

Wastewater from the rubber industry was selected for the experiments. Removal efficiency analysis was performed as stated by the standard methods for examination of water and wastewater.^29^ Filtered wastewater samples (100 mL) were poured into a dialysis tube; different bioflocculant concentrations (20, 40, and 60 mg L^−1^) were added and stirred with a magnetic stirrer for 15 min at 100 rpm, slowly stirred for 5 min at 50 rpm, and allowed to settle for 15 min. The volume of the supernatant was filtered through a filter paper and used for heavy metal analysis before and after bioflocculant treatment through inductively coupled plasma mass spectrometry (AAS; Model AA 6300, Shimadzu, Japan); the metal removal percentage rate of each element was also calculated.^30^ Controls (5 mL of deionized water) were also prepared in the dialysis tube for different metal-salt solutions. Removal percentage (*R*) was expressed as eqn (2).

### 2.6 Statistical analysis

Statistical analyses for the data collected were analysed using SPSS statistical version 25 software. Minimum three measurements were made to detect variability and to avoid biases. One-way ANOVA, a multiple comparison *post-hoc* test, was used for the determination of differences in each assay for cases of equality of variance assumed or not assumed. Significant differences were analysed through analysis of variance ANOVA for the basis of making conclusion and predication.^31,32^

## 3. Results and discussion

### 3.1 Characterization of bioflocculant QZ-7

#### 3.1.1 Qualitative analysis

The dried and purified bioflocculant product was whitish with a powdery form. Chemical analysis indicated the constituent total carbohydrates, proteins, and uronic acid chromogenic reaction, as shown in Table 1. The major components of the bioflocculant QZ-7 were polysaccharides, proteins, and uronic acid.

**Table 1:** Qualitative analysis of bioflocculant QZ-7

Component type	Analytical method	Occurrence
Polysaccharide	Anthrone test	+
Protein	Bradford test	+
Uronic acid	Carbazole-sulfuric acid test	+

#### 3.1.2 Fourier-transform infrared spectroscopy (FTIR)

**Fig. 1:**
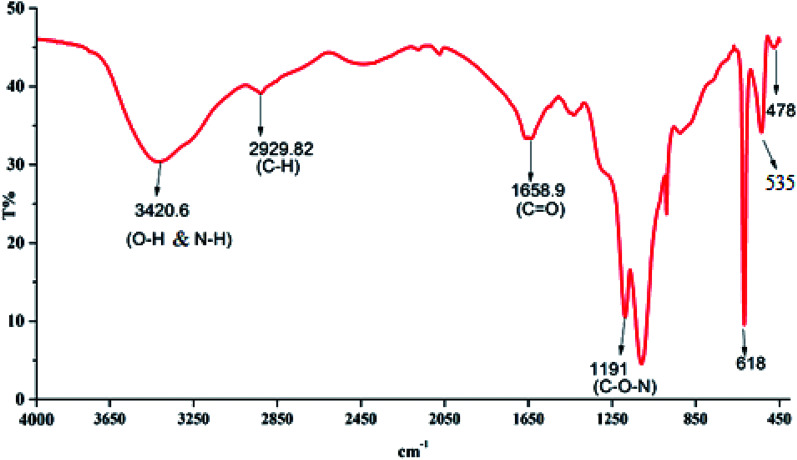
Fourier-transform infrared spectra.

The ability of a bioflocculant to capture pollutants not only depends greatly on molecular weight but also on the available functional groups, especially some charged ionic groups.^33,34^ Therefore, the FTIR spectra analysis of the bioflocculant in this study showed the presence of functional groups related to sugars and proteins. As shown in Fig. 1, a strong absorption peak is observed at 3420.56 cm^−1^ generated by the stretching vibration of the hydroxyl (–OH) or amino (–NH) groups. In contrast, the maximum band at 2929.82 cm^−1^ reflected the stretching vibration in the CH_3_ groups, while the stretching band of –CH_2_ was found at 2437.35 cm^−1^.^35^ The absorption peak at 1658.90 cm^−1^ corresponded to the presence of carbonyl groups, indicating a characteristic vibration of the C=O stretching in the –CONH group in proteins and amino sugars.^36^ The band numbers between 1187 and 900 cm^−1^ indicated significantly different sugar derivatives.^37^ The band at 1109.66 cm^−1^ was ascribed to the asymmetrical stretching of the C–O–C ester linkage. The presence of a β-glycoside bond between the sugar monomers was indicated by small absorption bands at 618.50 cm^−1^, 535.79 cm^−1^ and 478.32 cm^−1^. The presence of –OH, NH–, COOH– and COO– groups in the bioflocculant molecules is important for determining the flocculating activity, while the H^+^ and OH^−^ groups on the surface of the suspended particles may form hydrogen bonds when the bioflocculant chains approach the surface of the particles.^38,39^

#### 3.1.3 Scanning electron microscopy

**Fig. 2:**
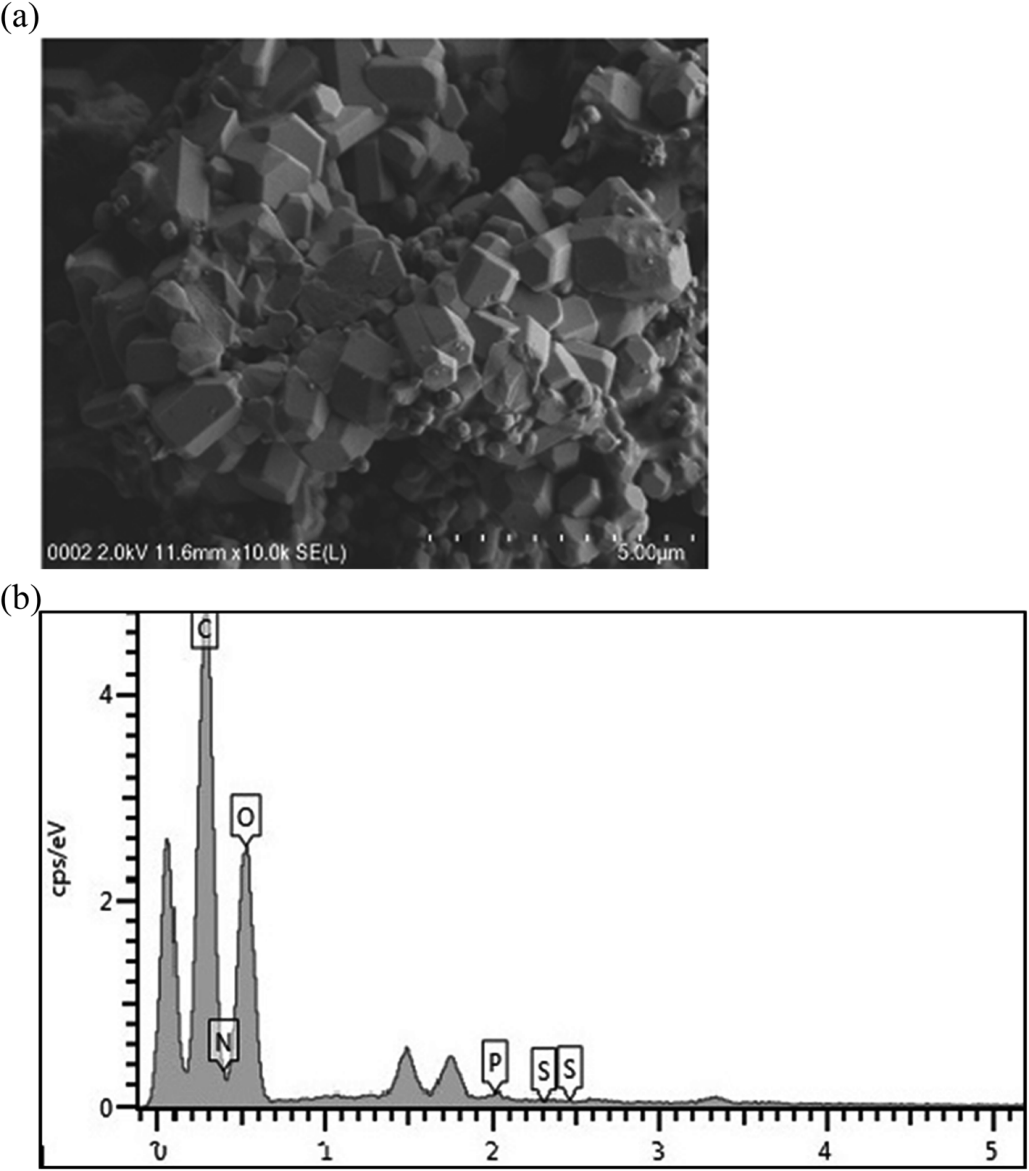
(a) SEM micrograph of bioflocculant QZ-7. (b) EDAX of bioflocculant QZ-7.

*B. salmalaya* strain 139SI produces an extracellular metabolite, that is, bioflocculant QZ-7. The SEM image in Fig. 2a shows the brick-shaped structure of bioflocculant QZ-7.

#### 3.1.4 SEM-energy dispersive X-ray

Fig. 2b shows the elemental analysis of the bioflocculant on the basis of SEM-EDX testing. C, O, N, P, and S in this macromolecule accounted for 55.74%, 42.74%, 0.54%, 0.93%, and 0.06%, of the bioflocculant, respectively. Salehizadeh *et al.*^40^ reported the presence of phosphorus and sulfur in low quantities in a bioflocculant. The bioflocculants containing sulfur are a perfect substrate for lead (Pb^2+^) adsorption because the ions possess high affinity towards many heavy metals including Pb^2+^.^41^

#### 3.1.5 Thermogravimetric determination of purified QZ-7

**Fig. 3:**
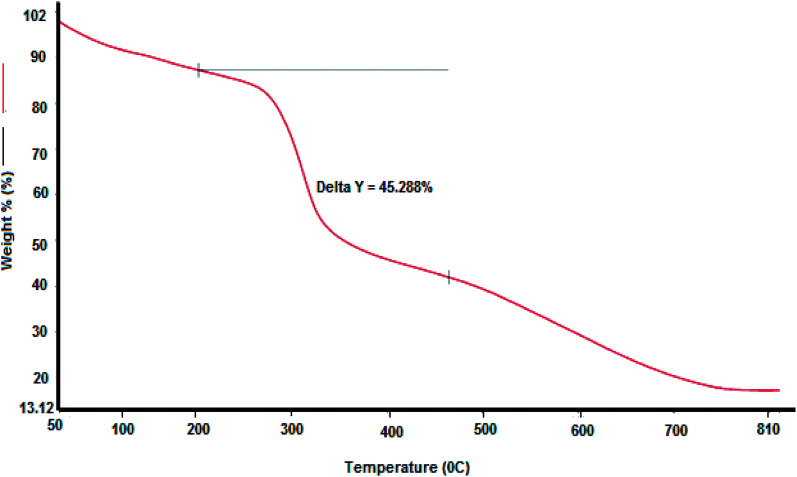
Thermogravimetric analysis of the purified QZ-7.

The bioflocculant QZ-7 was tested to elucidate its thermogravimetric behavior at different temperatures.^42^ The results are depicted in Fig. 3 to assist in recognizing its pyrolysis property after exposure to significantly elevated temperatures. Fig. 3 shows a weight loss of QZ-7 (%) under the effect of temperature between 50 and 800 °C. The maximum weight loss (61.13%) of QZ-7 was reported at 480 °C, while QZ-7 was completely pyrolyzed at a temperature higher than 480 °C; the weight loss was approximately 5% at 100 °C, 12.14% at 200 °C, and 25% at 300 °C. TGA of the bioflocculant QZ-7 exhibited thermo-labile and thermo-stable molecular contents, showing the combination of sugar and protein substances, as indicated by examination.

The first weight decrease may be due to the loss of available moisture or the presence of carboxyl and hydroxyl groups in the protein portions related to the glycoprotein-like molecules of QZ-7. Analogous observations were reported by Wang *et al.*^16^ with respect to the bioflocculant gained from the mixed consortium of bacteria.

### 3.2 Factors affecting flocculating performance of bioflocculant

#### 3.2.1 Effect of pH

**Fig. 4:**
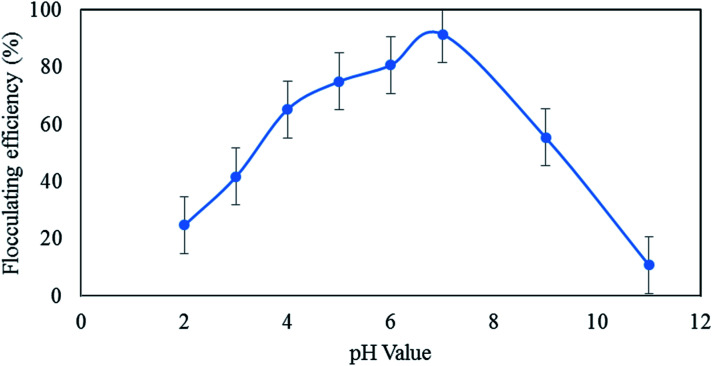
pH stability of the purified bioflocculant QZ-7.

Bioflocculant QZ-7 has a relatively wide pH tolerance ranging from acidic to slightly alkaline conditions. As shown in Fig. 4, the bioflocculant QZ-7 was found to be fairly steady at a wider pH range of 4–7, and over 70% flocculating activity was observed at this pH range with significant different performances (*p* < 0.00). As shown in Table 2, the highest flocculating activity was achieved at pH 7 with a mean difference of about 91.5% from the lowest activity measured at pH 2–3 and 11 (*p* < 0.05). Consequently, multiple comparison analysis revealed that pH 5 and pH 6 have the same effect on the flocculating activity of QZ-7.

**Table 2:** Analysis of variance for the effect of pH on flocculating performance

	Sum of squares	df	Mean square	F	Sig.
Between groups	16 405.203	7	2343.600	2014.895	0.000
Within groups	18.610	16	1.163		
Total	16 423.813	23			

#### 3.2.2 Effect of bioflocculant dosage

**Fig. 5:**
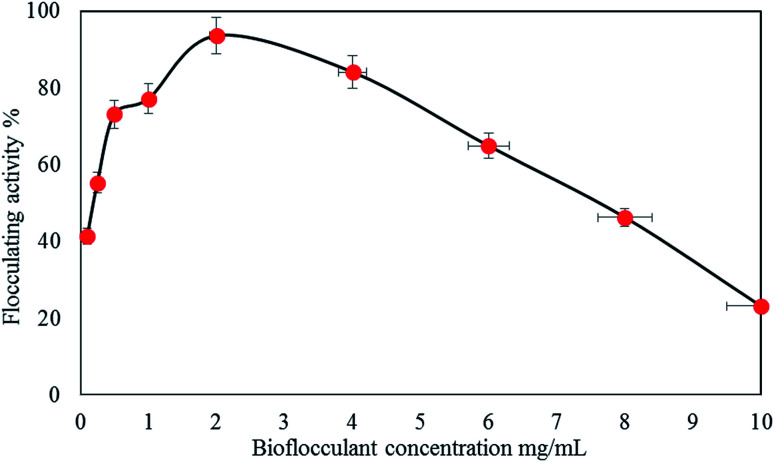
Bioflocculant QZ-7 dosage effect on flocculating activity.

The bioflocculant dosage concentration is one of the dynamic parameters deliberated during the investigation of the optimum conditions in coagulation–flocculation development.^43^ The performance in flocculation decreases due to the inadequate dose of a bioflocculant.^43^ Consequently, the bioflocculant optimal dosage should be set up to support the reduction of costs and achieve a better performance in treatment processes. In this section, a kaolin suspension was used to determine the optimal bioflocculant dosage for heavy metal removal. Fig. 5 shows the correlation between bioflocculant dosage and kaolin suspension removal. The bioflocculant concentration ranged between 0.1 mg mL^−1^ and 6 mg mL^−1^. The maximum removal value for kaolin suspension (93.66%) was reported at 2 mg mL^−1^ of the bioflocculant; decrease in turbidity may be caused by the adsorption of excess bioflocculant on the colloidal surfaces, causing colloid destabilization and thus blocking the available sites on the surface of particles for the improvement in the interparticle bridges.^44^ In contrast, the removal was reduced at a bioflocculant dosage higher than 2 mg mL^−1^. Gong *et al.*^45^ reported that an inadequate dosage of a bioflocculant used during treatment can lead to a poor bridging phenomenon, thus resulting in low flocculation activity.

Table 3 shows that the result obtained is in accordance with the reported findings for the flocculation performance of different concentrations of bioflocculants with significant differences (*p* < 0.00).

**Table 3:** Analysis of variance for the effect of bioflocculant QZ-7 concentration

	Sum of squares	df	Mean square	F	Sig.
Between groups	12 241.856	8	1530.232	834.503	0.000
Within groups	33.007	18	1.834		
Total	12 274.863	26			

#### 3.2.3 Cation effects on flocculation efficiency

**Fig. 6:**
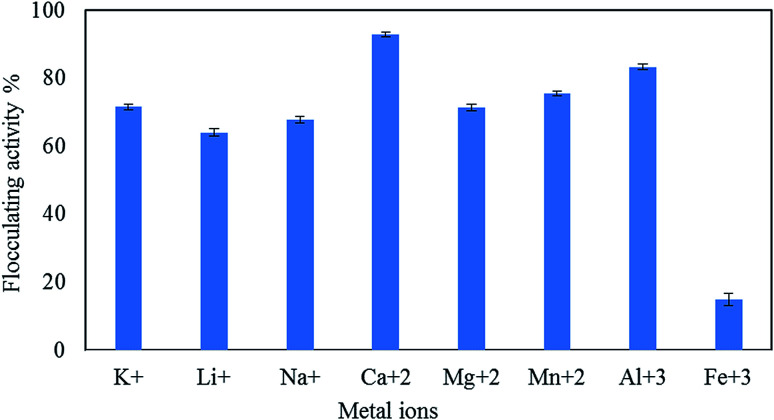
Metal ion effect on the flocculating activity of pure QZ-7.

Cations improved the coagulation–flocculation process of neutralizing and destabilizing the negatively charged residues of bioflocculant functional groups through linking particle bonds to the kaolin suspension.^46^ For instance, Fig. 6 shows that monovalent and divalent cations, including Al^3+^, can stimulate the flocculating activity to a significant extent compared to trivalent Fe^3+^ cations. The maximum flocculating efficiency (92.6%) was detected for Ca^2+^, followed by Al^3+^ (83.3%), Mn^2+^ (75.6%), K^+^ (71.6%), Mg^2+^ (71.4%), Na^+^ (67.7%), and Li^+^ (64%). These results are analogous to those reported by Okaiyeto *et al.* (2013), Wang *et al.* (2011), and Zheng *et al.* (2008).^16,46,47^ They reported that various cations, such as Ca^2+^, Mn^2+^, and Al^3+^, increased the flocculation activity of the bioflocculants produced by *Micrococcus* sp. and *Halomonas* sp., xn11 and xn7, and the mixed culture of *Bacillus sphaeicus* F6 and *Rhizobium radiobacter* F2. The bioflocculant produced by aquatic bacteria, *Oceanobacillus* sp., which was the most stimulated by cations, such as calcium chloride and aluminum chloride, was also detected.^48^ The bioflocculants produced by *Bacillus* sp. and *Virgibacillus* sp. were interactive, increasing the flocculating activity under the influence of Ca^2+^, Mg^2+^, and Mn^2+^.^48,49^ Calcium ions were more active and improved the formation of larger flocs when compared with other cations. Therefore, calcium ions were selected as the coagulant support for further experiments.

The statistical analysis presented in Table 4 indicates that there is a significant difference (*p* < 0.000) between different cations.

**Table 4:** Analysis of variance for the effect of cations on flocculating activity

	Sum of squares	df	Mean square	F	Sig.
Between groups	11 327.947	7	1618.278	1016.387	0.000
Within groups	25.475	16	1.592		
Total	11 353.422	23			

### 3.3 Application of bioflocculant QZ-7

#### 3.3.1 Influence of pH on heavy metal adsorption

**Fig. 7:**
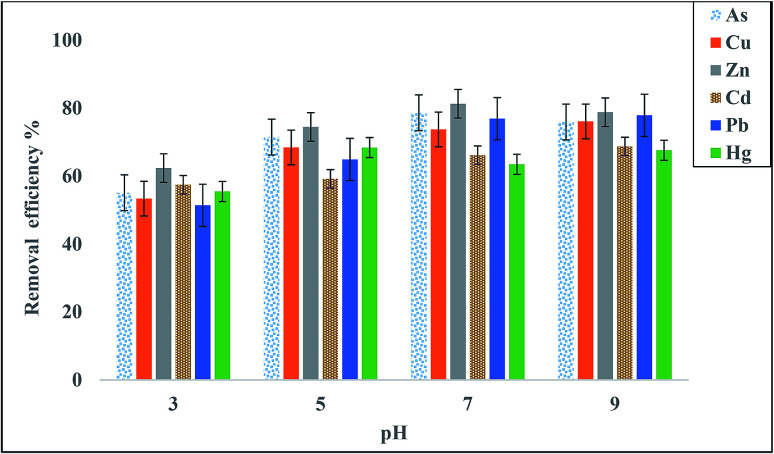
Effect of pH on heavy metal adsorption using 20 mg L^−1^ bioflocculant QZ-7.

pH is recognized as the most essential parameter due to the significant changes in pH that occur during heavy metal sorption. pH is directly linked to the interaction competency of hydrogen ions with the metal ions on the active sites of the biosorbent surfaces.^50^ Spectroscopic analysis (FTIR) indicated that the pure bioflocculant QZ-7 possessed an assortment of functional groups, including carboxyl, hydroxyl, and amino groups, which are virtually intricate in terms of binding mechanism potentials.^22^ These functional groups contribute to metal-ion binding and are dependent on the pH values of the aqueous solution. The influence of pH on heavy metal adsorption, such as those of As, Cd^2+^, Cu^2+^, Pb^2+^, Zn^2+^, and Hg^2+^, by the bioflocculant QZ-7 was investigated at pH 3–9. In Fig. 7, the heavy metal adsorption shows an increasing trend when the pH value is increased from 3 to 9, which is similar to the study of Dobrowolski *et al.*^35^ The highest adsorptions of Zn^2+^ and As were reported at pH 7, whereas the highest adsorptions of Cu^2+^, Pb^2+^, and Cd^2+^ were reported at pH 9. The highest adsorption of Hg^2+^ occurred at pH 5. Regarding the analysis of variance of the effect of different pH values on heavy metal removal efficiency, the results are presented in Table 5. It was revealed that different pH values have a significant difference (*p* < 0.00). The adsorption of heavy metals was high at neutral and alkaline pH values, and high proton concentrations at acidic pH values competed for the same anionic sites of the polymer as divalent cations. Proton mass contributes to the preferred binding, resulting in low divalent cation binding.^51^ For instance, the increase in pH towards the optimal value, which contrasted from one metal ion to another, and the saturated superficial adsorption by negative charges led to increased efficiency of positive charges to bind and adsorb metal ions.^52^ At a pH higher than their optimal value, hydroxide metals can be formed, and the adsorption sites on the surface fail to bind the adsorbent.^53^

**Table 5:** Analysis of variance for the effect of pH on arsenate removal efficiency

	Sum of squares	df	Mean square	F	Sig.
Between groups	2555.898	6	425.983	274.912	0.000
Within groups	21.693	14	1.550		
Total	2577.591	20			

#### 3.3.2 Effect of bioflocculant QZ-7 concentration on heavy metal adsorption

**Fig. 8:**
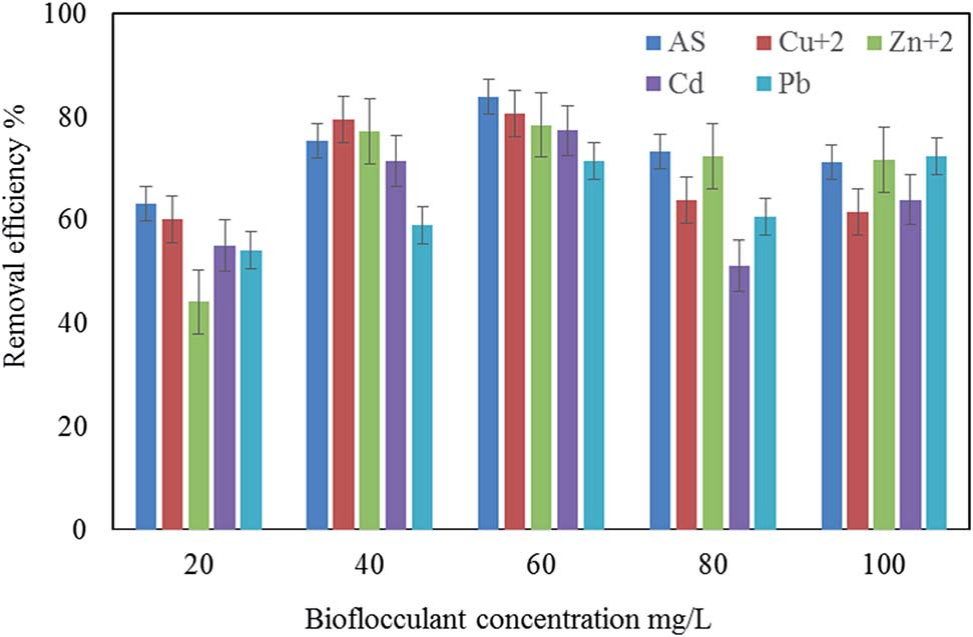
Effect of bioflocculant QZ-7 concentration on the removal efficiency of heavy metals.

The results in Fig. 8 show that the bioflocculant displayed better efficiency in heavy metal removal. As shown in Fig. 8, although the maximum removal for As (81%), Cu^2+^ (84%), Cd^2+^ (77%), Zn^2+^ (78.5%) and Pb^2+^ (71.5%) was reported at 60 mg L^−1^ of the bioflocculant concentration, significant removal for heavy metals (As (63%), Cu^2+^ (60%), Cd^2+^ (55%), Zn^2+^ (44%) and Pb^2+^ (54%)) was also obtained at a lower concentration of the bioflocculant (20 mg L^−1^). Similar results were also reported by Das and Santra.^54^ The increased heavy metal removal efficiencies at low bioflocculant concentrations will significantly enhance the development of industrial effluent wastewater treatment. Besides, in accordance with the multiple analyses shown in Table 6, there is a significant difference (*p* < 0.05) for different QZ-7 concentrations on heavy metal removal efficiency.

**Table 6:** Analysis of variance for the effect of QZ-7 concentration on the removal efficiency of heavy metals

	Sum of squares	df	Mean square	F	Sig.
Between groups	740.568	5	148.114	35.041	0.000
Within groups	50.723	12	4.227		
Total	791.291	17			

#### 3.3.3 Effect of metal concentration on removal efficiency using 60 mg L^−1^ of bioflocculant QZ-7

**Fig. 9:**
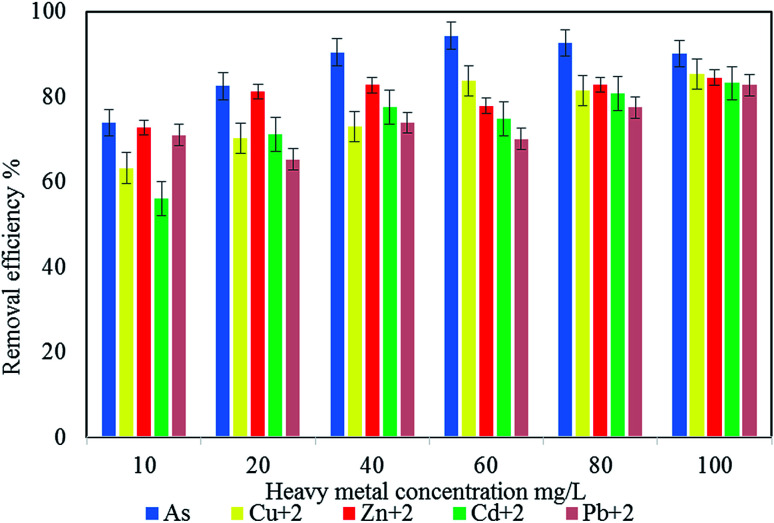
Effect of metal concentration on the efficiency of the removal of heavy metals.

The results presented in Fig. 9 show that heavy metal adsorption increased with increasing initial concentrations between 10 and 100 mg L^−1^, as indicated by the maximum removal of As (94.3%) at 60 mg L^−1^ and the optimum removal of Cu^2+^ (85.2%), Cd^2+^ (84.5%), Zn^2+^ (84.5), and Pb^2+^ (82.76%) at 100 mg mL^−1^. The improvement in metal adsorption may be due to the increase in electrostatic interactions and the constantly low affinity of the linking sites for metal ions.^55^ Hence, the presence of sulfur in a bioflocculant enhances its heavy metal capacity and makes it an excellent substrate for applications in heavy metal sequestration.^56^

Based on the multiple analyses of the effect of heavy metal concentration on the bioflocculant adsorption efficiency shown in Table 7, there is a significant difference (*p* < 0.05) for the different concentrations of heavy metals.

**Table 7:** Analysis of variance for the effect of heavy metal concentration on QZ-7 adsorption efficiency

	Sum of squares	df	Mean square	F	Sig.
Between groups	563.465	5	112.693	26.520	0.000
Within groups	50.991	12	4.249		
Total	614.457	17			

### 3.4 Removal of heavy metals from industrial wastewater using bioflocculant QZ-7

**Fig. 10:**
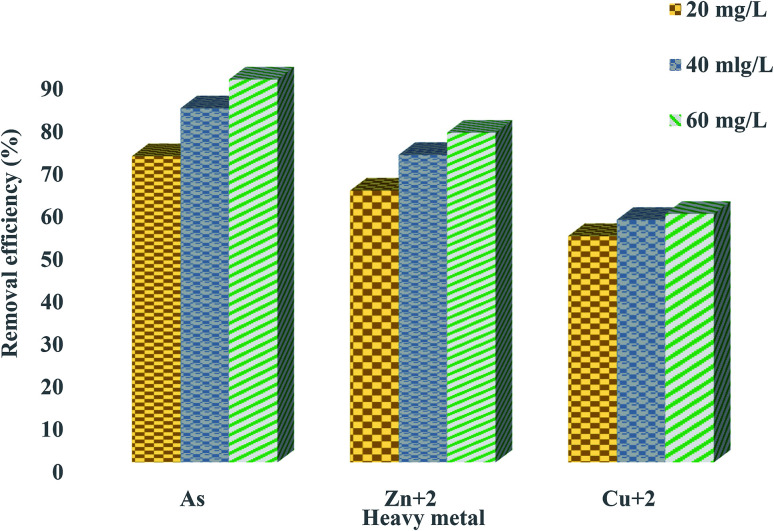
Removal efficiency of heavy metals using 20 mg L^−1^, 40 mg L^−1^ and 60 mg L^−1^ of bioflocculant QZ-7.

Rapid initial adsorption of the heavy metals can be achieved by the affinity action of amino, carboxyl and hydroxyl groups present in a bioflocculant.^30^ The treatment of industrial effluents with the bioflocculant QZ-7 at different concentrations, *i.e.*, 20, 40, and 60 mg L^−1^ showed effective flocculation with concomitant reduction in heavy metals. Similarly, Fig. 10 depicts the effect of adsorbent dosage on the adsorption of heavy metals by bioflocculant QZ-7. For example, As removal increased from 71.9% to 89.85% with the amount of adsorbent concentration, whereas Zn^2+^ removal also increased from 63.85% to 77.4%. On the other hand, Cu^2+^ removal slightly increased from 53.2% to 58.4%.

## 4. Conclusion

The present study investigated the performance of a new polymeric bioflocculant QZ-7 for the removal of heavy metals from industrial wastewater. The removal efficiency for As^3+^, Cu^2+^, Zn^2+^, Cd^2+^, and Pb^2+^ was evaluated under varying experimental conditions, such as pH, incubation time, initial concentration of metal ions, and adsorbent dosage. Maximum flocculating efficiency (92.9%) was achieved for Ca^2+^ at pH 7.0 ± 0.2 followed by Al^3+^ (83.3%); Mn^2+^, K^+^, and Mg^2+^ (over 70%); Na^+^ and Li^+^ (above 64%). The maximum adsorptions of Zn^2+^ (81.2%) and As (78.6%) were detected at pH 7, whereas the maximum removal of Pb^2+^ (77.9%), Cu^2+^ (76.1%), and Cd^2+^ (68.7%) was obtained at pH 9. The results showed that the bioflocculant QZ-7 can be applied for the removal of heavy metals from industrial wastewater within the concentration range from 20 mg L^−1^ to 60 mg L^−1^. The bioflocculant was effective in the removal of As, Zn^2+^, and Cu^2+^. The findings established that the efficiency of heavy metal removal depends on the dose of low-cost adsorbent and bioflocculant concentrations. The optimum pH range for heavy metal adsorption ranged from 7 to 9.

## Funding

This study was supported by the University of Malaya, UMRG Programme under Grant No. RP023A-14 AFR.

## Conflicts of interest

There are no conflicts to declare.

## Supplementary Material
